# The timing and precision of action prediction in the aging brain

**DOI:** 10.1002/hbm.23012

**Published:** 2015-10-27

**Authors:** Nadine Diersch, Alex L. Jones, Emily S. Cross

**Affiliations:** ^1^ Wales Institute of Cognitive Neuroscience, School of Psychology Bangor University Gwynedd United Kingdom; ^2^ Aging and Cognition Research Group German Center for Neurodegenerative Diseases (DZNE) Magdeburg Germany; ^3^ Department of Psychology Gettysburg College Pennsylvania; ^4^ Behavioural Science Institute and Donders Institute for Brain, Cognition, and Behaviour, Radboud University Nijmegen Nijmegen The Netherlands

**Keywords:** action anticipation, action observation network, caudate, fMRI, older adults, task performance

## Abstract

Successful social interactions depend on the ability to anticipate other people's actions. Current conceptualizations of brain function propose that causes of sensory input are inferred through their integration with internal predictions generated in the observer's motor system during action observation. Less is known concerning how action prediction changes with age. Previously we showed that internal action representations are less specific in older compared with younger adults at behavioral and neural levels. Here, we characterize how neural activity varies while healthy older adults aged 56–71 years predict the time‐course of an unfolding action as well as the relation to task performance. By using fMRI, brain activity was measured while participants observed partly occluded actions and judged the temporal coherence of the action continuation that was manipulated. We found that neural activity in frontoparietal and occipitotemporal regions increased the more an action continuation was shifted backwards in time. Action continuations that were shifted towards the future preferentially engaged early visual cortices. Increasing age was associated with neural activity that extended from posterior to anterior regions in frontal and superior temporal cortices. Lower sensitivity in action prediction resulted in activity increases in the caudate. These results imply that the neural implementation of predicting actions undergoes similar changes as the neural process of executing actions in older adults. The comparison between internal predictions and sensory input seems to become less precise with age leading to difficulties in anticipating observed actions accurately, possibly due to less specific internal action models. *Hum Brain Mapp 37:54–66, 2016*. © **2015 The Authors Human Brain Mapping Published by Wiley Periodicals, Inc.**

## INTRODUCTION

From skilled movements, like a penalty kick in football, to everyday actions, like shaking hands, anticipating what other people will do next is fundamental for successful social interactions. According to the predictive coding framework, incoming sensory signals are matched with internally generated predictions that reflect an observer's model of the world to infer the causes of sensory input [Clark, 2013; Friston, 2005]. During action observation, the observer's motor system is assumed to predict the sensory consequences of the observed action based on prior expectations about the most likely action goal [Friston et al., 2011; Kilner et al., 2007]. Mismatches between these two sources of information produce a prediction error signal that is used to adjust the internal action representation, which in turn changes the internal prediction. In line with this, it has repeatedly been shown that action observation engages frontoparietal regions of the human brain that may contain mirror neurons, which fire both when an action is executed and when the same or similar action is observed [Grosbras et al., 2012; Rizzolatti and Craighero, 2004]. Together with the posterior superior temporal sulcus (pSTS), they form the so‐called Action Observation Network (AON) [Grafton, 2009]. Studies in humans show that activity in these regions might not only support inferring the goal of an observed action, but also the prediction of others' actions [e.g., Balser et al., 2014; Bischoff et al., 2012; Cross et al., 2013; Fontana et al., 2012; Kilner et al., 2004; Urgesi et al., 2010; Wright et al., 2010]. In one such study, Cross et al. [2013] used an action occlusion paradigm to directly compare neural activity during action observation and the time when participants had to predict how the action continued while being occluded (i.e., no agent or action was visible on the screen). Watching the action sequences preferentially engaged brain regions implicated in visuospatial attention, whereas predicting them resulted in increased activity in the AON. At the neurophysiological level, it was shown that mirror neurons in the premotor cortex (PMC) of the macaque monkey also discharge when final parts of an observed action are hidden [Umilta et al., 2001]. Maranesi et al. [2014] recently confirmed that a subset of PMC mirror neurons show predictive rather than reactive discharge during action observation in a predictable context.

Less is known about functional changes in this network as the brain ages. Generally, older compared to younger adults show less selectivity in relevant neural networks and recruit additional regions during performance of a variety of tasks [Grady, 2012; Park and Reuter‐Lorenz, 2009]. It follows that an age‐related de‐differentiation in the motor system might be one reason for altered representations of observed actions [cf., Carp et al., 2011; Heuninckx et al., 2005; Léonard and Tremblay, 2007; Seidler et al., 2010]. On the sensory side, some evidence suggests that older adults are less efficient in inferring changes in the environment. Older adults might have problems in adapting their internal models in response to sensory input that is novel or violates prior expectations [cf., Vieweg et al., 2015]. For example, Moran et al. [2014] characterized age‐related changes in functional connectivity in an auditory mismatch negativity paradigm and showed that rapid sensory updating decreases with age as evidenced by an age‐related reduction of bottom‐up prediction error signals during the processing of oddball relative to learned stimuli. Thus, problems in updating internal action representations in response to sensory input might further constrain the ability to accurately predict others' actions in old age.

At the behavioral level, we previously found that older compared to younger adults predict the time‐course of observed actions less precisely, although task performance in both age groups benefited from sensorimotor experience in the observer [Diersch et al., 2012]. Prediction performance was assessed using an action occlusion paradigm in which participants observed different action sequences that were partly occluded and whose continuation after occlusion was shifted towards the past or the future, or was temporally congruent. Participants' task was to judge the temporal coherence of the action continuation. In a follow‐up functional magnetic resonance (fMRI) study, we studied changes in brain activity during action prediction in older and younger adults while using a simplified version of the paradigm from the behavioral study with two types of action sequences that differed in their degree of motor familiarity [Diersch et al., 2013]. With respect to age, the results showed that older adults recruit regions beyond the AON, which younger adults did not, during the prediction of partly occluded actions. However, the inclusion of two action categories resulted in a limited number of different continuations after occlusion and prediction trials, which prohibited us from examining several critical questions, including: (1) whether neural activity is modulated by the temporal shift of the continuation after occlusion (e.g., depending on whether the action continuation is shifted backwards or forwards in time); (2) whether correct and erroneous prediction trials on incongruent continuations are processed differentially; and (3) whether a lower sensitivity in predicting the time‐course of the observed actions is linked to specific neural activation patterns in older adults. Here, we aimed to bridge this gap and to determine for the first time the extent to which brain activity is related to the temporal shift of the action continuation after occlusion and the ability to predict ongoing actions in old age. A group of healthy older adults underwent fMRI while observing a subset of the video stimuli used in our previous studies consisting of action sequences that were highly familiar to the observers and proved to engage the AON. The actions were partly occluded at critical time‐points and continued after occlusion either congruently in time, or too early or too late on two different levels resulting in five possible continuations after occlusion. Participants were asked to indicate whether the continuation was too early or too late. Neural activity was measured at the beginning of the occlusion in an event‐related design and age and psychophysical indices for the timing and sensitivity of prediction were integrated in fMRI data analyses.

In line with previous research, we expected the prediction of partly occluded actions to engage brain regions typically involved in predictive processing of others' actions, such as frontoparietal and occipitotemporal cortices of the AON [cf., Bubic et al., 2010; Diersch et al., 2013]. We further hypothesized that the temporal shift of the continuation after occlusion should modulate activity in the AON. If the AON is specifically tuned to predict ongoing actions and is used to generate internal predictions about the most‐likely action trajectory during the occlusion period, th**e**n activity within this network should be greater the earlier in time the actions continue after occlusion (i.e., representing action states that happened during the occlusion period) [cf., Cross et al., 2013]. However, the AON's capability to support the extrapolation of actions into the future when sensory input is lacking might be limited, especially in the aging observer. If this were the case, we would expect that the more the continuations after occlusion represent future action states, the more activity might shift to regions beyond the AON. This assumption would be further supported by differences in neural activity between correct and erroneous predictions that are expected to vary between too early and too late continuations after occlusion. More specifically, action continuations that are shifted toward the future might erroneously be judged as being too early if they are still predominantly processed within the AON. One might also argue that neural activity measured at the beginning of occlusion reflects the mismatch between the internal prediction and sensory input (i.e., prediction error signal). In this case, AON activity might increase the more the temporal shift of the observed continuation deviates from the congruent continuation. With respect to differences in neural activity on the between‐subject level, we expected increasing age to be associated with activity increases in sensory and prefrontal cortices similar to findings from studies investigating the processing of executed actions in the aging brain [e.g., Heuninckx et al., 2005; Seidler et al., 2010]. Based on findings suggesting that the caudate modulates AON engagement in conditions of higher uncertainty, we further hypothesized that caudate activity should relate to individual participants' sensitivity in action prediction [Diersch et al., 2013; Schiffer and Schubotz, 2011].

## MATERIAL AND METHODS

### Participants

After being screened for health status and potential contraindications to MRI scanning, 24 older adults were invited to take part in the fMRI experiment. Exclusion criteria included a history of any major physical or neurological disease, psychiatric disorder, and use of medication that might affect cognitive performance or blood flow. Participants provided written informed consent and were paid for their participation in accordance with the local ethics committee. Three participants were excluded from data analyses due to problems with following task instructions during scanning. The final sample consisted of 21 healthy older adults (12 female, mean age: 64.3 ± 4.28 years, age‐range: 56–71 years) with normal or corrected‐to‐normal vision. They were right‐handed (LQ = 92.9 ± 8.32); [Oldfield, 1971] and showed no signs of cognitive decline as measured by means of the Mini‐Mental State Examination score = 29.1 ± 0.83 [Folstein et al., 1975]. Participants reported 14.6 ± 3.19 years of education on average and all of them were highly familiar with the observed actions. On average, participants rated their current as well as their past ability to execute the observed actions as being “good” (*M* = 2.29 ± 0.65 vs. *M* = 1.65 ± 0.65) on a 5‐point rating scale ranging from “very good” to “very bad.” Their ratings about their past performance ability were more positive than those about their current ability, *t*_(20)_ = 6.04, *P* < 0.001.

### Experimental Stimuli and Procedure

**Figure 1:**
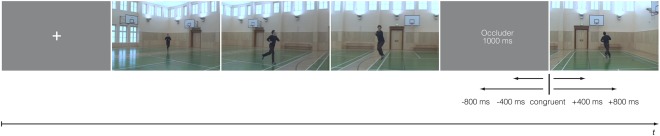
Trial sequence during fMRI scanning: After fixation (1000 ms), participants observed different action sequences showing highly familiar movement exercises that were occluded for 1000 ms at critical time‐points. After occlusion, the videos continued congruently, temporally too early or too late on two different levels (±400/±800 ms). [Color figure can be viewed in the online issue, which is available at http://wileyonlinelibrary.com.]

During fMRI scanning, participants observed different action sequences consisting of simple movement exercises (e.g., knee bend, jumps, spins, running forwards or backwards, and step sequences such as alternating spinning and running) that were performed by a female and male non‐athlete in a sports hall. The duration of the videos was 9.0 s on average (range: 8.0–10.9 s). A prediction trial started with a fixation cross (1000 ms), followed by the beginning of an action sequence. The actions were visible for 4.5 s on average (range: 3.1–5.9 s) before they were occluded for 1000 ms by a grey rectangle. The continuation after the occlusion was either congruent, temporally too early or too late on two different levels (±400/±800 ms, Fig. 1). Participants were instructed to judge the temporal coherence of each observed action continuation by pressing one of two response keys with their index or middle finger on a response device placed in the right hand (left key: too early, right key: too late) while the remainder of the action sequence was shown. They were not informed that the actions might continue congruently on some trials. The action sequences were presented in blocks each consisting of eight videos, in which no action was repeated consecutively. Continuations after occlusion were randomized separately with the restriction that not more than three too early, congruent, or too late continuations and a maximum of two identical continuations were presented in a row. After eight action sequences, a black screen with a grey fixation cross was presented for 8–12 s in a pseudo‐randomized order that served as baseline condition. Order of the action sequences and continuations after occlusion were counterbalanced across participants. In total, participants performed 160 prediction trials across two functional runs (8 action sequences × 2 actors × 5 continuations after occlusion × 2 repetitions).

At the day of the health screening, participants also practiced the prediction task with a subset of videos not used in the fMRI experiment. This training consisted of 64 trials (duration: 15 min) during which the temporal coherence of the four incongruent continuations (−800, −400, +400, and +800 ms, 16 trials per continuation) had to be judged. Participants received feedback of their performance. Because participants were always forced to decide whether the actions continued too early or too late, the congruent continuation was not presented at this stage due to a lack of a correct response option. Time between the training and fMRI scanning was 43.3 ± 28.5 days on average.

### fMRI Data Acquisition

Scanning was performed on a 3T Philips Achieva scanner with a SENSE 8‐channel phased‐array head coil. In two functional runs, 459 whole‐brain EPI images were acquired in ascending order (918 in total) with a repetition time of 2000 ms (30 axial slices). Imaging parameters were as follows: echo time, 30 ms; slice thickness, 4mm; no interslice gap; in‐plane resolution, 3 mm × 3 mm; acquisition matrix, 76 × 75 voxels; and flip angle, 90°. High‐resolution anatomical images were acquired using a T1‐weighted 3D magnetization‐prepared rapid gradient echo (MPRAGE) sequence with the following parameters: repetition time, 12 ms; echo time, 3.5 ms; flip angle, 8°; image matrix, 240 × 240 voxels; spatial resolution, 2 × 2 × 2 mm^3^.

### Behavioral Data Analysis

In addition to a standard analysis of variance (ANOVA) on the proportion of too early responses with continuation after occlusion as repeated‐measures variable, the timing and sensitivity in action prediction was analyzed psychophysically. By fitting a psychometric function over the z‐transformed too early response rates on every continuation after occlusion by means of a linear regression, two psychophysical indices could be derived: (1) the point of subjective equality (PSE), which is the point in time at which participants judged the action continuation on chance level, that is, at which they perceived it as being just‐in‐time (i.e., the prediction timing) and (2) the just‐noticeable difference (JND), which represents the difference that is reliably noticed in half of the cases (75% − 25%)/2 or the steepness of the psychometric function (i.e., the prediction sensitivity) [cf., Gescheider, 1997].

### fMRI Preprocessing and Data Analysis

Image preprocessing and statistical analyses were performed in SPM8 (Wellcome Department of Imaging Neuroscience, London, UK). Preprocessing of the EPI volumes included realignment, slice timing, and spatial normalization to MNI space [Ashburner and Friston, 2005]. All volumes were spatially smoothed using an 8 mm full‐width half‐maximum Gaussian kernel. Statistical analyses were based on the generalized linear model (GLM) approach. In the first GLM, neural activity was modeled at the beginning of occlusion for each continuation after occlusion (−800 ms, −400 ms, congruent, +400 ms, and +800 ms) on the individual level as separate events with 0 s duration convolved with the standard hemodynamic response function to capture the neural response during the occlusion period in which participants were assumed to internally predict the occluded action sequences, in line with previous research using action occlusion paradigms [cf., Cross et al., 2013; Diersch et al., 2013; Stadler et al., 2011]. Each baseline was modeled as a boxcar with the respective duration. The time of the button press was modeled as a nuisance variable as were six realignment parameters obtained from motion correction to control for the effects of movement during action prediction. A temporal high‐pass filter of 128 s was applied to remove low‐frequency scanning artifacts. The effects of continuation after occlusion compared with baseline were computed for each participant and tested on the group level by means of one‐sample *t*‐tests. We further checked for areas where activity changed as a function of the temporal shift from the earliest to the latest continuations and vice versa by using two linear contrasts (+2, +1, 0, −1, −2 and −2, −1, 0, +1, +2) over continuations after occlusion (−800 to +800 ms). Changes in neural activity as a function of temporal distance to the congruent continuation were examined by means of two additional polynomial contrasts (−4, +1, +6, +1, −4 and +4, −1, −6, −1, +4) over continuations after occlusion. This allowed us to examine the extent to which AON engagement is modulated by the temporal shift of the continuation after occlusion, either depending on whether the action continuation is shifted backwards or forwards in time or depending on the extent of deviation from the correct continuation after occlusion.

In a second GLM, correct and incorrect predictions on incongruent continuations after occlusion were separately modeled (too early/correct, too early/error, too late/correct, and too late/error). This allowed us to test whether erroneous predictions might be associated with different neural activation patterns than correct predictions on too early and too late continuations after occlusion. Each of these regressors contained both levels of temporal delay (±400/±800 ms) to ensure that a reasonable number of valid events enter the analysis. All the other regressors were modeled in the same way as in the first GLM.

In additional analyses, the relation between neural responses and between‐subject variability was examined in more detail. Therefore, the individual contrast images for the comparison between prediction and baseline from the first GLM were entered into a multiple regression analysis. Values for the timing in action prediction (i.e., PSE values), the sensitivity in action prediction (i.e., JND values) that were based on a psychometric function fitted across responses on the five continuations after occlusion, and, given the large age range in our sample, the age of the observer served as predictor variables. This allowed us to identify the unique contribution of each source onto neural activation patterns while ensuring that both the neural measure (i.e., the BOLD contrast) and the behavioral indices share the same basis, that is, responses across every continuation after occlusion. The sex of the observer was additionally included as covariate of no interest due to a correlation between the JND and sex observed at the behavioral level.

To take into account our a priori hypotheses about the relation between neural activity and between‐subject variability, anatomical regions of interest (ROI) were defined for regions consistently implicated in action observation (the AON) [Grafton, 2009] and found to be activated during the observation of dynamic whole‐body movements in a recent ALE meta analysis [Grosbras et al., 2012; see also Caspers et al., 2010]. These included the lateral occipito‐temporal cortex (corresponding to visual area MT/V5), the pSTS, the anterior inferior parietal lobule (IPL; corresponding to BA40), the inferior frontal gyrus (IFG; corresponding to BA44/BA45), and the lateral and medial PMC (corresponding to BA6). Based on the results of our previous study, we additionally checked whether activity in the caudate is related to the sensitivity in action prediction [cf., Diersch et al., 2013]. The single ROIs were created based on each participant's T1 structural scan using a semiautomated anatomic reconstruction and labeling procedure as implemented in FreeSurfer v5.3.0, which has been shown to be comparable in terms of accuracy to manual labeling techniques (http://surfer.nmr.mgh.harvard.edu) [Dale et al., 1999; Fischl et al., 1999]. In each hemisphere, labels corresponding to the IFG (BA44/45), PMC (BA6), anterior IPL (supramarginal gyrus as defined in the Desikan‐Killiany Atlas), pSTS (banks of the superior temporal sulcus as defined in the Desikan‐Killiany Atlas), and MT produced by the automatic cortical parcellation as well as the caudate from the subcortical segmentation were extracted [Desikan et al., 2006; Fischl et al., 2002]. This procedure has the advantage of accounting for age‐related structural changes in the brain since it does not involve the mapping of different brains to a common atlas that refers to a young brain. Each ROI was thresholded at 0.5 and transformed to MNI space for the usage in SPM8. Finally, cluster ROIs representing the whole sample were built based on each of the individual ROIs, with the constraint that adjacent cortical ROIs such as the PMC and IFG, did not contain any overlapping voxels.

All analyses were conducted with a voxel‐level height threshold of *P* < 0.001 (uncorrected). Whole brain analyses focus on brain regions reaching a cluster‐corrected threshold of *P* < 0.05, family wise‐error (FWE) corrected. For the analyses of the effects of age and prediction performance on neural activity, we applied small volume corrections for the brain volume determined by the respective ROI (*P* < 0.05, FWE corrected). Anatomical localizations of significantly activated clusters were aided by the Anatomy Toolbox in SPM8 [Eickhoff et al., 2005] in combination with the Atlas of the Human Brain [Mai et al., 2008]. Significant activations are displayed on a mean image of the normalized T1 images of the sample and parameter estimates in corresponding peak voxels were extracted for illustrative purposes by using rfxplot [Glaescher, 2009].

## RESULTS

### Behavioral Results

**Figure 2:**
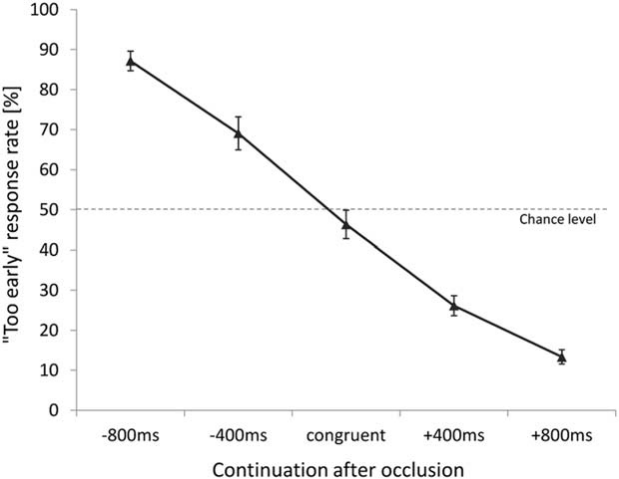
Too early response rates on every continuation after occlusion. Error bars denote standard errors of the means (SE).

An ANOVA on the proportion of too early responses with continuation after occlusion (−800 ms, −400 ms, congruent, +400 ms, +800 ms) as repeated‐measures variable revealed a significant main effect of continuation after occlusion, *F*_(4,80)_ = 195, *P* < 0.001. The proportion of too early responses declined linearly across time as indicated by a significant linear trend model, *F*_(1,20)_ = 924, *P* < 0.001 (Fig. 2). Performance was at chance level when a congruent continuation was observed, *t*_(19)_ = 1.04, *P* = .312, whereas for every incongruent continuation participants performed well above chance level, all *t* ≥ 4.63, *P* ≤ 0.001. Performance of the sample was not biased towards the past or the future as implied by a PSE of −29.3 ± 270 ms that did not differ significantly from zero, *t*_(20)_ = 0.50, *P* = 0.625. However, a relatively large time‐bin around the congruent continuation was predominantly perceived as being just‐in‐time (JND = 440 ± 105 ms).

The prediction timing and sensitivity were not related to the motor familiarity or age of the observer, as suggested by nonsignificant correlations between PSE or JND values and the age of the observer or experience with observed actions (all *r* ≤ .290, *P* ≥ 0.201, two‐tailed). This confirms that experience with the observed actions did not influence performance on the task, as all participants were highly familiar with the observed actions. Previously, we showed that the JND but not the PSE is affected by the age of the observer when comparing older and younger adults [Diersch et al., 2012]. Given that the current sample only encompassed older adults, the age range might have been too small to detect a reliable relation to prediction sensitivity. There was, however, a negative relation between the sex of the observer and the JND (*rho* = −0.553, *P* = 0.009, two‐tailed) implying that female observers of the sample showed a higher uncertainty about the time‐course of the action continuations than male observers. This might be attributable to a sampling bias in the present study since we did not observe similar effects in our previous studies [Diersch et al., 2012, 2013]. To control for potentially confounding effects of sex in the present data, sex is included as a covariate of no interest when the analysis of the fMRI data takes the observers' level of prediction sensitivity (JND) into account.

### fMRI Results

**Figure 3:**
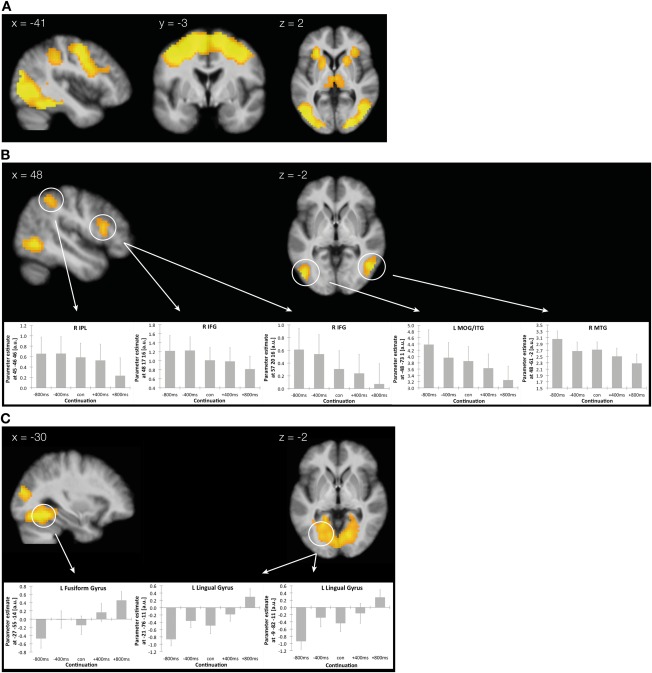
**A:** Overall effects of predicting occluded actions collapsed across continuations after occlusion compared with baseline. Linear changes in neural activity across continuations after occlusion: **B:** Activity increases the earlier in time the actions continued after occlusion and **C:** activity increases the later in time the actions continued after occlusion. Clusters are displayed on a mean image of the normalized T1 images of the sample. Results were calculated using a voxel‐wise threshold of *P* < 0.001. Only clusters are shown that reached a cluster‐corrected threshold of *P* < 0.05, FWE corrected across the whole brain. Plots depict parameter estimates of the respective peak voxels in each cluster per continuation after occlusion. Error bars indicate the across‐subject standard error of the mean. [Color figure can be viewed in the online issue, which is available at http://wileyonlinelibrary.com.]

First, we looked at the overall effects of predicting occluded actions irrespective of performance by comparing neural activity at the beginning of occlusion across every continuation after occlusion to the baseline condition. This analysis revealed significant bilateral activations in motion‐sensitive occipitotemporal regions and frontoparietal regions of the AON, including IPL, lateral PMC, and IFG. We also found significantly activated clusters in the bilateral medial PMC comprising supplementary motor area (SMA proper) and pre‐supplementary motor area (pre‐SMA), in the bilateral anterior insula extending to the basal ganglia with peaks in the putamen, the thalamus, and the brainstem (Fig. 3A, Table 1). No differences in neural activity were obtained for the comparison between congruent and incongruent continuations after occlusion and vice versa.

**Table 1:** Effects of predicting partly occluded actions

Contrast	Region	MNI coordinates (*x*, *y*, *z* in mm)	*Z* score (voxel level)	Cluster size
Prediction > baseline	L Middle Occipital Gyrus	−42, −82, −2	6.37	8375
	L Superior Frontal Gyrus (SMA)	−6, −1, 64	6.28	
	R Middle Temporal Gyrus	48, −61, −2	6.25	
	Brainstem	0, −31, −11	5.37	694
		0, −40, −41	4.64	
	R Thalamus	3, −16, 1	4.31	
	R Insula	33, 29, 4	4.81	207
	R Putamen	21, 11, 1	4.27	
Linear contrast:	R Middle Temporal Gyrus	48, −61, −2	5.20	173
Too early >Too late (+2, +1, 0, −1, −2)	L Middle Occipital Gyrus/Inferior	−48, −73, 1	5.34	135
	Temporal Gyrus			
	R Inferior Parietal Lobule	45, −46, 46	4.58	126
	R Inferior Frontal Gyrus	48, 17, 16	4.25	86
		57, 20, 16	3.83	
Linear contrast:	L Fusiform Gyrus	−27, −55, −14	5.73	2436
Too late >Too early (−2, −1, 0, +1, +2)	L Lingual Gyrus	−21, −76, −11	5.59	
		−9, −82, −11	5.58	

Spatial coordinates of the local maxima for the prediction of partly occluded actions compared to baseline, activity increases the earlier in time the actions continued after occlusion, and activity increases the later in time the actions continued after occlusion. Results were calculated using a voxel‐wise threshold of *P* < 0.001. Only clusters are shown that reached a cluster‐corrected threshold of *P* < 0.05, FWE corrected across the whole brain.

Next, we examined in which regions activity changed linearly as a function of the temporal shift of the continuation after occlusion by means of two linear contrasts. This analysis showed that activity in the right IFG and IPL together with the bilateral posterior temporal gyrus at the transition to the lateral occipital cortex (visual area MT/V5) increased the earlier in time the actions continued after occlusion (Fig. 3B, Table 1). The inverse direction of the contrast revealed increases in activity in early visual areas (visual areas V1 to V3) the later in time the actions continued after occlusion (Fig. 3C, Table 1). The analysis of possible changes in neural activity as a function of temporal distance to the congruent continuation did not reveal any supra‐threshold activations.

#### Performance‐related differences during the prediction of incongruent continuations after occlusion

**Figure 4:**
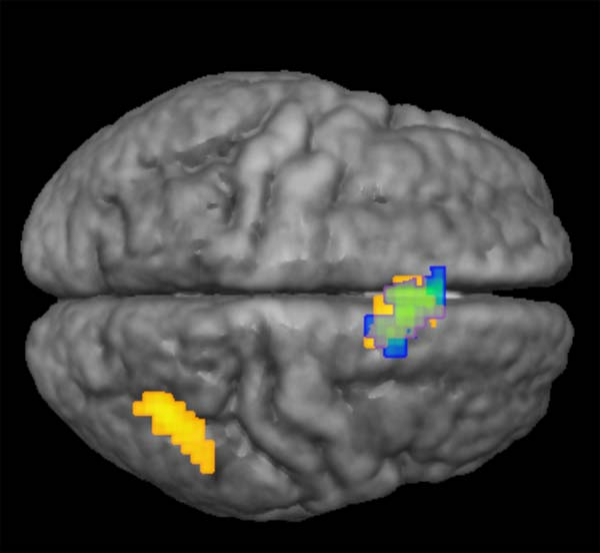
Differences in brain activity for incorrect predictions on too early continuations (blue) and incorrect predictions on too late continuations (orange) compared with correct too late predictions rendered on a dorsal view of the mean image of the normalized T1 images of the sample. Results were calculated using a voxel‐wise threshold of *P* < 0.001. Only clusters are shown that reached a cluster‐corrected threshold of *P* < 0.05, FWE corrected across the whole brain. [Color figure can be viewed in the online issue, which is available at http://wileyonlinelibrary.com.]

When comparing neural activity on incongruent (too early and too late) continuations after occlusion while taking only correctly predicted action sequences into account, a similar pattern was obtained as in the linear contrast analyses in the first GLM. For the comparison between correctly predicted too early continuations and correctly predicted too late continuations, enhanced activity in right IFG and IPL as well as in bilateral occipitotemporal cortices was found (Table 2). Supra‐threshold activations additionally included the medial and lateral PMC. For the inverse direction of the contrast, early visual cortices were more activated, similar to the corresponding linear contrast (Table 2). The comparisons between false predictions on too early versus too late continuations and vice versa did not reveal any activation differences that reached cluster‐corrected significance. Notably, when too early continuations that were incorrectly perceived as being too late were compared with correct too late predictions, increased activity in the pre‐SMA was observed (Fig. 4, Table 2). The pre‐SMA was also more involved when comparing incorrectly perceived too late predictions to correct too late predictions (Fig. 4, Table 2). For this direction of the contrast, the right IPL was additionally engaged.

**Table 2:** Effects of predicting partly occluded actions as a function of prediction performance on incongruent continuations after occlusion

Contrast	Region	MNI coordinates (*x*, *y*, *z* in mm)	*Z* score (voxel level)	Cluster size
Correct too early > correct too late	R Inferior/Middle Temporal Gyrus	54, −61, −5	5.82	697
	R Supramarginal Gyrus	45, −43, 43	5.11	
	R Inferior Parietal Lobule	39, −55, 46	4.34	
	R Inferior Frontal Gyrus	57, 20, 16	4.22	335
		48, 11, 22	4.14	
	R Middle Frontal Gyrus	39, 11, 49	3.96	
	L Inferior/Middle Temporal Gyrus	−51, −73, 1	5.59	215
	L Inferior Occipital Gyrus	−57, −46, 4	3.42	
	R Superior Frontal Gyrus (pre‐SMA)	3, 26, 46	4.09	78
Correct too late > correct too early	L Fusiform Gyrus	−27, −73, −11	5.68	1639
	L Lingual Gyrus	−12, −76, −8	5.54	
		−21, −49, −8	5.25	
Incorrect too early > correct too late	R Superior Frontal Gyrus (pre‐SMA)	−3, 17, 43	4.84	193
		15, 11, 58	3.59	
Incorrect too late >correct too late	R Angular Gyrus	39, −58, 40	4.25	104
	R Inferior Parietal Lobule	33, −52, 34	3.71	
		48, −52, 52	3.22	
	R Superior Frontal Gyrus (pre‐SMA)	3, 17, 52	3.81	95

Spatial coordinates of the local maxima for the comparison too early vs. too late continuations, too late vs. too early continuations that were correctly predicted, and too early continuations or too late continuations that were incorrectly predicted vs. correctly predicted too late predictions. Results were calculated using a voxel‐wise threshold of *P* < 0.001. Only clusters are shown that reached a cluster‐corrected threshold of *P* < 0.05, FWE corrected across the whole brain.

#### Between‐subject effects

**Figure 5:**
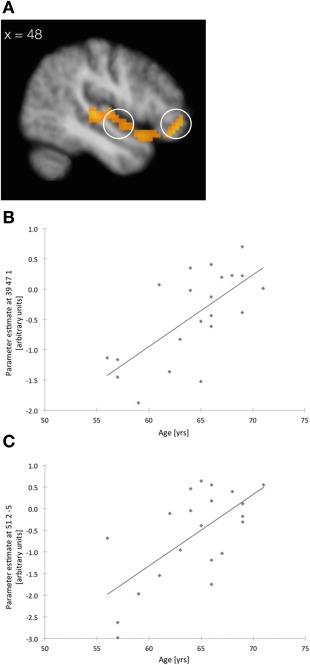
**A:** Regions in which activity covaried positively with the age of the observer. Results are displayed with a threshold of *P* = 0.005 (uncorrected) on a mean image of the normalized T1 images of the sample to show the subthreshold extent of the activated regions. Parameter estimates of the peak voxel in **B:** the frontal cortex and **C:** the anterior STG/STS plotted against the age of the observer. [Color figure can be viewed in the online issue, which is available at http://wileyonlinelibrary.com.]

To examine the relationship between neural responses and between‐subject variability, a multiple regression analysis was performed within predefined ROIs with values for the timing in action prediction (i.e., PSE), the sensitivity in action prediction (i.e., JND), and age as predictor variables while controlling for the sex of the observer. We additionally explored whether individual differences on these measures were associated with activity changes in regions elsewhere in the brain. Increasing age in our sample (aged 56–71 years) was associated with increasing activity in the anterior portion of the right IFG ROI that belonged to a larger cluster in the right middle frontal gyrus/frontopolar cortex when considering the entire brain (Fig. 5, Table 3). The ROI analysis further revealed two clusters in the right pSTS ROI that correlated positively with age reaching significance at the voxel‐level (*P* ≤ 0.041) and approaching significance when correcting for multiple comparisons at the cluster‐level (*P* ≤ 0.055, Table 3). When taking the whole brain into account, it was shown that these voxels formed part of a larger cluster with peaks in the anterior portion of right superior temporal gyrus (STG)/STS that were more activated the older the observer was (Fig. 5, Table 3). We did not find any region in which activity correlated negatively with age within the ROIs or elsewhere in the brain.

**Table 3:** Between‐subject effects

Contrast	Region	MNI coordinates (*x*, *y*, *z* in mm)	*Z* score (voxel level)	Cluster size	*P*‐value	*P*‐value
					(FWE corr., cluster‐level)	(FWE corr., voxel‐level)
ROI analysis: Age positive	R IFG	39, 47, 1	4.28	64	0.005	0.012
		45, 41, −8	4.21			0.015
		42, 44, −5	4.19			0.016
		36, 44, −14	3.92			0.040
	R pSTS	54, −43, −8	3.74	6	0.050	0.021
		51, −25, 1	3.59	5	0.055	0.032
		48, −28, 4	3.50			0.041
Whole‐brain analysis: Age positive	R Middle/Inferior Frontal Gyrus	39, 47, 1	4.28	80	0.036	
	R Inferior Frontal Gyrus	45, 41, −8	4.21			
		36, 44, −14	3.92			
	R Superior Temporal Gyrus	51, 2, −5	3.74	75	0.044	
	R Superior Temporal Sulcus	60, −4, −8	3.70			
	R Temporal Pole	51, 14, −14	3.64			
ROI analysis: JND positive	L Caudate	−6, 5, 4	3.25	4	0.049	0.064

Spatial coordinates of the local maxima during prediction vs. baseline that correlated positively with the age of the observer and the sensitivity in action prediction (JND) in a‐priori regions‐of‐interest and across the whole‐brain. Results were calculated using a voxel‐wise threshold of *P* < 0.001. *P*‐values refer to the FWE corrected significances at the cluster‐level for the a‐priori region‐of‐interest or the whole brain and FWE corrected significances at the voxel‐level for the a‐priori region‐of‐interest.

**Figure 6:**
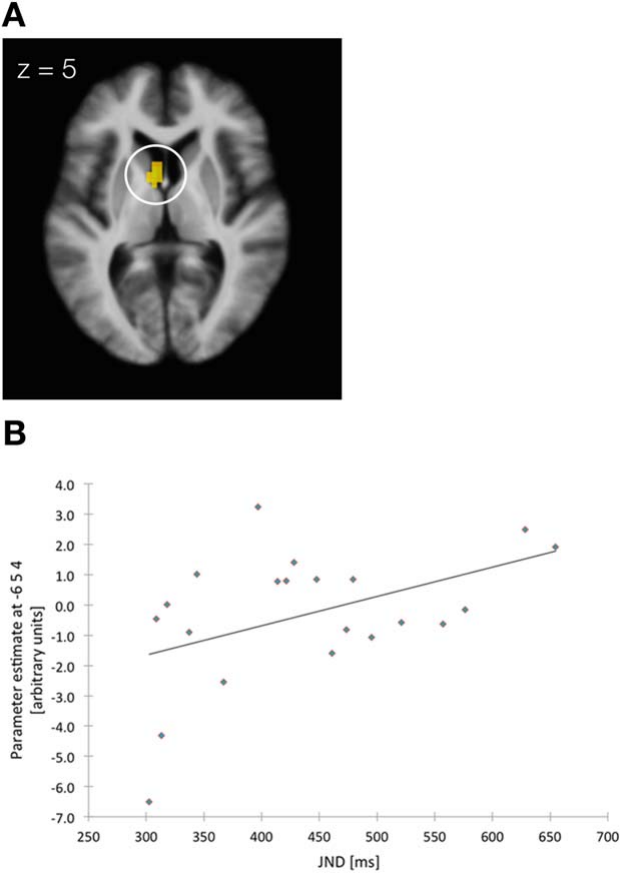
**A:** Regions in which activity correlated positively with the JND of the observer. Results are displayed with a threshold of *P* = 0.005 (uncorrected) on a mean image of the normalized T1 images of the sample to show the subthreshold extent of the activated region. **B**: Parameter estimates of the peak voxel in the caudate plotted against the JND of the observer. [Color figure can be viewed in the online issue, which is available at http://wileyonlinelibrary.com.]

We further examined whether a lower sensitivity in action prediction (i.e., higher JND) was associated with greater engagement of the caudate nucleus by means of a ROI analysis. In line with our hypothesis, we found that the amplitude of activity in the left head of the caudate ROI correlated positively with the JND of the observer (Fig. 6, Table 3). Activity in this region reached significance at the cluster‐level (*P* = 0.049) and was marginally significant when correcting for multiple comparisons at the voxel‐level (*P* = 0.064). There was no region in which activity correlated negatively with the JND. We did not observe any significant effects of the timing in action prediction (i.e., PSE) within the ROIs or elsewhere in the brain.

## DISCUSSION

The prediction of observed actions is less precise and is accompanied with more extended brain activations in older compared to younger adults [Diersch et al., 2012, 2013]. What has remained unclear is how neural activity varies while healthy older adults predict the time‐course of an unfolding action as well as the relationship between neural activity and the ability to predict observed actions as the brain ages. In line with previous research, we show that older adults engage brain regions commonly associated with predictive processing when they judge the time course of highly familiar action sequences that are partly occluded and whose continuation after occlusion is temporally manipulated [cf., Bubic et al., 2010; Cross et al., 2013]. Compared with baseline, action prediction resulted in increased activity in occipitotemporal and frontoparietal regions of the AON showing that the observed actions proved suitable to engage this network in the aging brain. However, one should note that this comparison most likely also contains more general aspects of the stimuli and task activation.

Crucially, neural activity was modulated by the point in time to which the action continuation after occlusion referred: the more the action continuation was shifted backwards in time, reflecting action states that happened during the occlusion period, the greater parts of the AON were engaged. Conversely, early visual cortices were more engaged the more the action continuation after occlusion was shifted towards the future. This pattern was also highly evident when incorrectly predicted action continuations were excluded from the analysis. This suggests that older adults, like younger adults, generate internal predictions about the most‐likely action trajectory with the help of their sensorimotor system during the occlusion period [cf., Cross et al., 2013]. Our results therefore extend findings from previous studies showing that mirror neurons in the PMC of the macaque monkey show predictive discharge [Maranesi et al., 2014; Umilta et al., 2001] and that brain regions composing the AON in the human brain are engaged when predicting others' actions [Fontana et al., 2012; Kilner et al., 2004; Urgesi et al., 2010]. Our results further imply that generating predictions about action states that would happen after the occlusion period seems to increasingly rely on the visual system. Thus, aging observers' capability to extrapolate partly occluded actions into the future within the AON appears to be limited. Previously, we argued that older adults process observed actions more visually, based on the finding that they engage early visual cortices more than younger adults during action prediction compared with baseline [Diersch et al., 2013]. The current findings highlight that this might depend on the point in time to which the continuation after occlusion refers. Age‐related increases in the recruitment of the visual system might relate to future action states in particular.

This is further supported by the observed differences in neural activity between correct and erroneous predictions that varied as a function of the temporal shift of the continuation after occlusion. We found that the pre‐SMA is generally more engaged when the actions continued too early after occlusion, irrespective of participants' judgements, compared with correctly predicted too late continuations. Higher activity in the pre‐SMA was also found when comparing incorrectly predicted too late continuations to correctly predicted too late continuations. In younger adults, the pre‐SMA has been associated with action monitoring and ordering movements in time [Nachev et al., 2008] and, during action prediction, with the maintenance of an internal action representation in general [Stadler et al., 2011]. Thus, maintaining an internal action representation within sensorimotor cortices did not seem to support performance in the aging observer when continuations were shifted beyond the occlusion period. The greater engagement of right IPL in addition to pre‐SMA for incorrect compared to correct too late predictions lends additional support to this notion.

Importantly, the time of occlusion as well as the amount of visual input after occlusion was the same between too early, congruent, and too late continuations. Differences in neural activation patterns therefore cannot be ascribed to differences in the amount of action information being presented. Moreover, brain activity did not vary as a function of the temporal deviation of the observed continuation from the congruent continuation. Given that each continuation after occlusion appeared equally often and participants were trained on the task beforehand, they expected the action continuations to be shifted in time. Thus, neural activity measured at the beginning of occlusion did not seem to reflect the mismatch between the internal prediction and sensory input (i.e., neural prediction error signal) but rather the neural implementation of action prediction.

### Between‐Subject Effects

Participants in our sample ranged in age from 56 to 71. We identified two areas in the brain where neural activity was positively correlated with the age of the observer, both of them in close vicinity to classical AON sites in the younger brain. Specifically, activity increases were found in anterior IFG that extended to the middle frontal gyrus/frontopolar cortex as well as in anterior STG/STS. This implies that neural activity becomes less specific and spreads from posterior IFG and STS to more anterior parts of these regions in older adults, which already becomes evident when considering an age range of 15 years beginning at about the age of 56. In younger adults, the anterior IFG has been implicated in encoding more abstract features of observed actions, such as their goal and intention [Press et al., 2012]. It has been suggested that once the most likely action‐goal is anticipated, a prediction about the sensory consequences of the action that is appropriate for achieving this goal might be encoded in the posterior IFG and compared with the actual sensory input [Kilner, 2011]. This is in agreement with a prominent model of lateral prefrontal cortex (PFC) functioning that proposes a rostro‐caudal hierarchical gradient of cognitive control [Koechlin et al., 2003]. Whereas posterior/caudal parts of the PFC are involved in selecting and updating action plans in response to relevant stimuli, more anterior/rostral parts seem to be driven by response uncertainty and track alternative courses of action, which constitute more abstract forms of cognitive control [Boorman et al., 2009; Levy and Wagner, 2011]. Less is known about the implications of variations in neural activity along the posterior‐anterior axis of the STS during action observation, although several studies report anterior in addition to posterior STS engagement while participants observe biological motion [e.g., Herrington et al., 2012; Puce and Perrett, 2003].

Interestingly, age‐related activity increases in regions such as the PFC are a common finding in the motor control literature [Seidler et al., 2010]. For example, Heuninckx et al. [2005] found that older compared with younger adults engage additional brain regions including the PFC, IFG, PMC, and the STG during execution of hand‐foot actions and argued that motor performance requires more cognitive control with advancing age [see also Heuninckx et al., 2008]. Our findings imply that the prediction of observed actions, which draws on similar neural resources as action execution in the younger brain, shows a similar pattern of change when the brain ages. Subsequent studies may now attempt to directly compare the neural processing of action prediction and action execution in older adults to provide further support for this assumption. Based on their finding that prediction error signals are attenuated in older adults in an auditory oddball paradigm, Moran et al. [2014] recently proposed that internal models get less complex with advancing age and consequently require less functional specialization in the brain. Thus, the activity increases in the IFG and STS might indicate that internal predictions might become less detailed with increasing age resulting in difficulties to capture slight temporal variations of the action continuations in our paradigm.

The only region that showed performance‐dependent activity changes in our sample was the caudate. The lower the sensitivity in action prediction, the higher was the BOLD response in this region. Caligiore et al. [2013] argued that basal ganglia‐cortical loops underlie the competition between different internal action models that are processed in parallel in the AON during action observation. This implies that basal ganglia engagement should be elevated in ambiguous conditions where none of the selected internal models matches well with the sensory input. In line with this, the caudate has been implicated in encoding deviations from initial predictions during action observation [Schiffer and Schubotz, 2011] and in predicting the time‐course of less familiar compared with highly familiar actions in younger adults [Diersch et al., 2013]. The present findings suggest that the caudate is similarly involved during the prediction of familiar actions when they are less precisely represented within the AON due to age‐related changes in brain function. Notably, age‐related deficits in motor adaptation when confronted with sensorimotor perturbations have been linked to striatal dysfunction in selecting appropriate representations after changes in the environment [King et al., 2013]. Marchand et al. [2011] found that functional connectivity between the caudate and sensory or motor cortices increases with age and is negatively correlated with performance in a motor learning task. One might therefore assume that age‐related deficits in predicting observed actions are linked to functional connectivity changes in neural networks processing prediction error signals during action observation.

## CONCLUSION

Here, we show for the first time that neural activity in the AON of healthy older adults during the prediction of partly occluded actions is sensitive to the time point to which the action continuations after occlusion refer. We further found that neural activity becomes less distinct in parts of the AON between 56 and 71 years of age, whereas lower prediction sensitivity is linked to increases in striatal activity. Similar changes have been observed in studies examining the neural processing of executed actions in older adults. In line with current models of age‐related changes in brain function based on the predictive coding framework, the results suggest that ability to accurately predict actions performed by others declines with advancing age as a result of less specific internal action models and difficulties in minimizing prediction error signals in response to sensory input.
